# T158. THE VALUE OF ACTIGRAPHY FOR MEASURING APATHY IN PATIENTS WITH SCHIZOPHRENIA: ASSOCIATIONS WITH CLINICAL MEASURES AND NEUROIMAGING OF ACTION INITIATION

**DOI:** 10.1093/schbul/sby016.434

**Published:** 2018-04-01

**Authors:** Marie-José van Tol, Claire Kos, Michelle Servaas, Jan Bernard Marsman, Nicky Klaasen, Oliver Tucha, Henderikus Knegtering, André Aleman

**Affiliations:** University of Groningen

## Abstract

**Background:**

Apathy is a highly debilitating and frequently occurring behavioral characteristic, present in approximately half of the patients with schizophrenia. Apathy is considered a core negative symptom that relates strongest to lack of initiative and is the strongest predictor of poor functional outcome, poor medication compliance, and high caregiver burden. Notwithstanding, measuring apathy is still a challenge. Therefore, we aimed to investigate whether actigraphy, an objective and continuous measurement of activity levels, could yield an objective quantitative measure of apathy severity in schizophrenia patients. Moreover, we aimed to investigate whether actigraphy related to relevant functional neuroimaging underpinnings of apathy, i.e. self-initiated goal-directed behavior.

**Methods:**

Quantity, variability, and initiation of motor behavior were studied in relation to apathy severity as measured with clinical measures, and in relation to neural correlates of self-initiated behavior using functional Magnetic Resonance Imaging (fMRI). All patients (N=58) suffered from clinical significant apathy, as measured with the Apathy Evaluation Scale and Scale for the Assessment of Negative Symptoms and wore an actigraph for 48 continuous hours. Physical activity was quantified as the total activity counts over the patients’ ten most active hours of each day, summed over two weekend days (Activity-total, i.e. 20 hours in total). Variability of motor behavior (Activity-variability) was calculated by taking the root of the Mean Squared Successive Difference of the activity counts. For 31 of these patients, fMRI data during a task tapping into self-initiative was available.

**Results:**

Results showed that quantity, variability, and initiation of motor behavior were associated with negative symptoms, but not specifically with apathy. Motor behavior parameters were associated with brain activation during the self-initiative task in various brain regions including inferior parietal regions. The results were only observed during the condition wherein participants were asked to promptly reply to specific cues and not during the condition where more freedom in timing and selection of behavior was allowed.

**Discussion:**

Actigraphy can be used to measure quantity as well as variability of motor behavior in patients with schizophrenia and with specific relevance for negative symptoms, and that it correlates with selective neural substrates of action selection and activation of motor programs. However, actigraphy may not capture higher-order motivational processes that contribute to apathy severity.

